# Adult Plant Development in Triticale (× *Triticosecale* Wittmack) Is Controlled by Dynamic Genetic Patterns of Regulation

**DOI:** 10.1534/g3.114.012989

**Published:** 2014-09-01

**Authors:** Tobias Würschum, Wenxin Liu, Katharina V. Alheit, Matthew R. Tucker, Manje Gowda, Elmar A. Weissmann, Volker Hahn, Hans Peter Maurer

**Affiliations:** * State Plant Breeding Institute, University of Hohenheim, 70599 Stuttgart, Germany; † Crop Genetics and Breeding Department, China Agricultural University, 100193 Beijing, China; ‡ ARC Centre of Excellence for Plant Cell Walls, University of Adelaide, Waite Campus, Urrbrae SA 5064, Australia; § Saatzucht Dr. Hege GbR Domäne Hohebuch, 74638 Waldenburg, Germany

**Keywords:** dynamic QTL, plant development, genetic architecture, triticale, complex traits, genome-wide association study (GWAS), high-density genotyping, Multiparent Advanced Generation Inter-Cross (MAGIC), multiparental populations, MPP

## Abstract

Many biologically and agronomically important traits are dynamic and show temporal variation. In this study, we used triticale (× *Triticosecale* Wittmack) as a model crop to assess the genetic dynamics underlying phenotypic plasticity of adult plant development. To this end, a large mapping population with 647 doubled haploid lines derived from four partially connected families from crosses among six parents was scored for developmental stage at three different time points. Using genome-wide association mapping, we identified main effect and epistatic quantitative trait loci (QTL) at all three time points. Interestingly, some of these QTL were identified at all time points, whereas others appear to only contribute to the genetic architecture at certain developmental stages. Our results illustrate the temporal contribution of QTL to the genetic control of adult plant development and more generally, the temporal genetic patterns of regulation that underlie dynamic traits.

Many traits of biological or agronomic importance are dynamic traits that change with time (Wu and Lin 2006). Consequently, a static examination at only a single time point neglects these temporal changes and can therefore only deliver a simplified view of genetic control in time. With the availability of high-density genotypic data, approaches to unravel the genetic architecture underlying important traits have become feasible (Würschum 2012; Edwards *et al.* 2013) and have been applied in different crops, including maize (*e.g.*, Buckler *et al.* 2009; Steinhoff *et al.* 2012), wheat (*e.g.*, Reif *et al.* 2011a), barley (*e.g.*, Wang *et al.* 2012), sugar beet (*e.g.*, Würschum *et al.* 2011), and rapeseed (*e.g.*, Snowdon *et al.* 2010; Würschum *et al.* 2013). Even for dynamic traits, however, most genomic approaches so far have been based on a single evaluation of the trait, and few studies have employed dynamic quantitative trait loci (QTL) mapping. Yan *et al.* (1998) performed QTL mapping for plant height in rice at several developmental stages and found that some QTL could be detected at all examined stages whereas others were only identified at some of the developmental stages. Busemeyer *et al.* (2013a) and Liu, *et al.* (2014) recently have described QTL mapping for biomass yield in triticale. They assessed three different time points and showed that the entire genetic architecture underlying biomass accumulation is under dynamic genetic control. Nevertheless, due to the low number of studies that use dynamic QTL mapping, our understanding of the genetic patterns underlying phenotypic plasticity remains rudimentary.

Adult plant development is an important trait in small grain cereals because it affects adaptation and ultimately yield. Most studies until now have investigated only the genetics underlying a single developmental stage, mainly flowering time. However, the genetic control underlying preceding or subsequent developmental stages may well be different. Winter cereals are sown in autumn and after vernalization during winter can make the transition from vegetative to generative growth, dependent on external stimuli such as photoperiod. After flowering, the grains start to fill and the plants finally ripen in summer. To enable a uniform coding of phenologically identical growth stages of crops, Lancashire *et al.* (1991) introduced a decimal system, the BBCH code. It is based on the well-known code for cereals developed by Zadoks *et al.* (1974) and is widely used by scientists and breeders alike.

Association mapping often is carried out on a diversity panel of lines but also can be used for QTL detection in multiple segregating families (Yu *et al.* 2008; Liu *et al.* 2013). Combining multiple families in a mapping population can offer the advantage of an increased QTL detection power. Triticale (× *Triticosecale* Wittmack) is a hexaploid crop containing the A and B genomes from wheat and the R genome from rye. QTL mapping in multiple families of triticale recently has been reported for biomass yield and plant height (Alheit *et al.* 2014).

The objective of this study was to investigate the genetic patterns of regulation underlying adult plant development in triticale. To this end, a large mapping population of 647 doubled haploid (DH) lines derived from four families was scored for developmental stage at three time points, roughly corresponding to the stage when awns become visible, late flowering, and very early dough development. In particular our objectives were to identify main effect QTL for the three developmental stages, assess the contribution of epistasis to the genetic architecture, and to investigate the temporal changes in the genetic control underlying phenotypic plasticity of adult plant development.

## Materials and Methods

### Plant material, field trials, and phenotypic data

The plant material and the field trials underlying this study have been described in Busemeyer *et al.* (2013a). In brief, a mapping population consisting of 647 DH (Würschum *et al.* 2012a) triticale lines was used. The population consisted of four families (Supporting Information, Figure S1), DH06 (*n* = 131; parents ‘Modus’ × ‘Saka3006’), DH07 (*n* = 120; parents ‘Modus’ × ‘Saka3008’), EAW74 (*n* = 200; parents ‘HeTi117-06’ × ‘Pawo’), and EAW78 (*n* = 196; parents ‘HeTi117-06’ × ‘TIW671’), which have been described by Alheit *et al.* (2011, 2012). These four families are partially connected because families DH06 and DH07 share a common parent (‘HeTi117-06’) as well as families EAW74 and EAW78 (‘Modus’) (Figure S1). This design was chosen because the six parents are all adapted elite winter triticale lines, which obliterates the need for a NAM design (Buckler *et al.* 2009). Furthermore, we wanted to include six parental lines to increase the number of alleles segregating in the mapping population and at the same time wanted to study large families. Fully connected designs, for example, a diallel (Rebaï and Goffinet 1993; Blanc *et al.* 2006) would have required a strongly increased size of the mapping population and consequently the partially connected design was chosen.

The DH lines were grown in partially replicated designs (Williams *et al.* 2011), including common checks with 960 yield plots per location, at two locations in 2 yr. The plants were scored visually for their developmental stage (*i.e.*, BBCH stage; Lancashire *et al.* 1991) at three time points termed DS1, DS2, and DS3, in May and June, at which time the majority of the plants were at the developmental stages: BBCH stage 49 (awns visible), BBCH 69 (late flowering), and BBCH 81 (very early dough development). Scoring was done on a plot basis, and the values represent the developmental stage of the majority of the plants within the plots. For all lines, the best linear unbiased estimates were determined across environments.

### Association mapping in multiple families

The DH lines were genotyped with DArT markers, and QTL mapping was done based on the integrated consensus linkage map described by Alheit *et al.* (2012). For QTL mapping, an additive genetic model was chosen, and association mapping was done with a biometric model that performed well in a recent comparison of models for association mapping in multiple families and incorporates a family effect to control for differences in family means, cofactors to control the genetic background, and a marker effect across families (Liu *et al.* 2012; Würschum *et al.* 2012b):

$$Y=l\mu +X_{f}M_{f}+X_{q}^{}{\text{b}}_{q}^{}+\sum_{c\neq q}X_{c}^{}{\text{b}}_{c}^{}+\text{ε}$$

In this model, **Y** is a *N* × 1 column vector of the best linear unbiased estimates of *N* DH lines, coming from *F* families (*F* = 4); **l** is a *N* × 1 column vector containing constant 1; *µ* is the intercept; **X*_q_*** (**X*_c_***) is a *N* × 1 column vector containing the marker information of each DH at marker *q* (cofactor *c*); **X*_f_*** is a *N* × *F* matrix whose elements are 1 or 0 according to whether or not an individual belonged to family *f* and **M*_f_*** is a *F* × 1 vector of family effects; b*_q_* (b*_c_*) is the expected allele substitution effect of marker *q* (cofactor *c*); and ε is the vector of the residuals of the model.

We applied a two-step procedure for QTL detection. In the first step, stepwise multiple linear regression was used to select a set of cofactors based on the Schwarz (1978) Bayesian Criterion with a model including a family effect and cofactors. Cofactor selection was performed using Proc GLMSELECT implemented in the statistical software SAS (SAS Institute Inc, 2008). In the second step, we calculated a *P* value for the association of each marker with the phenotypic value for the *F* test with a full model (with marker effect) against a reduced model (without marker effect). The Bonferroni-Holm procedure (Holm 1979) was used to detect markers with significant (*P* < 0.05) main effects. QTL were declared as overlapping between the three developmental stages if they fell within an arbitrarily defined 10-cM interval surrounding the QTL. The proportion of the genotypic variance explained by the detected QTL was estimated following Utz *et al.* (2000) as *p_G_* = $R_{adj}^{2}$ / *h^2^*. The *p_G_* explained by individual QTL (Table S2 and Table S3) was estimated as sequential partial *R^2^* from a simultaneous fit of the detected QTL. To account for the potential presence of colinearity among the detected QTL, they were fitted in the linear model in the order of the strength of their association with the trait, *i.e.*, the most significantly associated markers (smallest *P* value in the QTL scan) were fitted first in the model. Fivefold cross-validation was done as described previously (Liu *et al.* 2013; Würschum and Kraft 2014).

For the detection of epistasis the model was extended and included the main effects of the two loci *q* and *q*’, **X*_q_***b*_q_* and **X*_q_***_**’**_b*_q_*_’_, and the interaction effect of the marker pair under consideration **X*_qq_***_**’**_b*_qq_*_’_. For the significance level for the epistatic QTL we used an α-level of 0.05 and followed the suggestion of Holland *et al.* (2002) dividing the α-level by the number of possible independent pairwise interactions between chromosome regions, assuming two separate regions per chromosome (*P* < 5.3e-5). The circular plots illustrating the epistatic interactions were created with Circos (Krzywinski *et al.* 2009).

## Results

**Figure 1:**
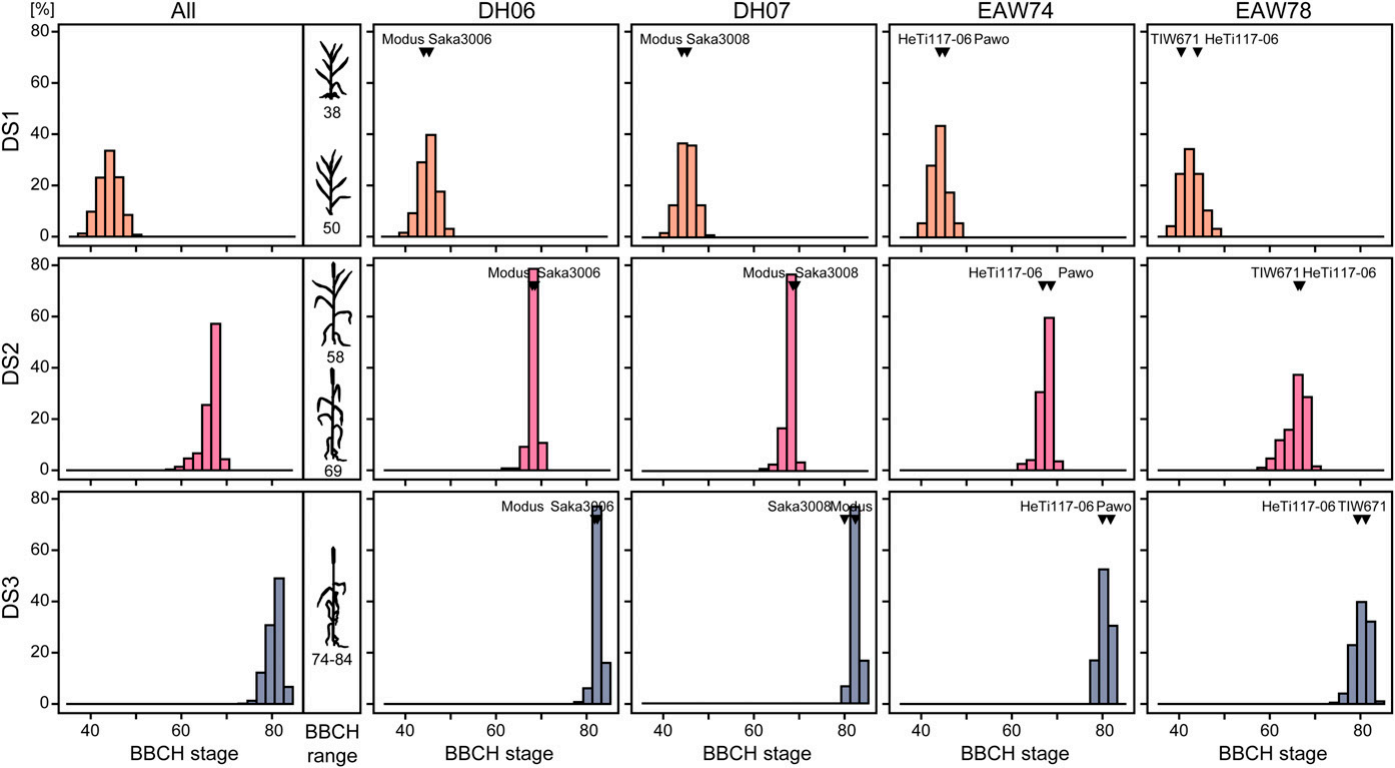
Histograms of the phenotypic values for developmental stage (BBCH) shown for the entire population and for each of the four families at three time points (DS1−DS3). The arrowheads indicate the parents of the families.

For all four families and developmental stages there were only small differences between the phenotypic values of the parental lines (Figure 1). The parents are all adapted elite winter triticale lines and the differences between families were marginal compared with the variation within families. In each of the four families DH lines transgressed the respective parents for all three time points and consistently, we observed highly significant (*P* < 0.01) genotypic variances ($\sigma_{G}^{2}$) and in addition genotype-by-environment interaction variances ($\sigma_{G\times E}^{2}$) for developmental stage at all three time points (DS1–DS3) (Table S1). The heritabilities in the triticale mapping population with 647 genotypes were high with 0.90 for DS1, 0.85 for DS2, and 0.72 for DS3. The phenotypic correlations between developmental stage at the three time points were 0.84 for DS1−DS2, 0.74 for DS2−DS3, and 0.69 for DS1−DS3 (all significant at *P* < 0.01). Taken together, the phenotypic data capture the developmental progression over time and the data set is therefore well suited to study the underlying genetic dynamics.

**Figure 2:**
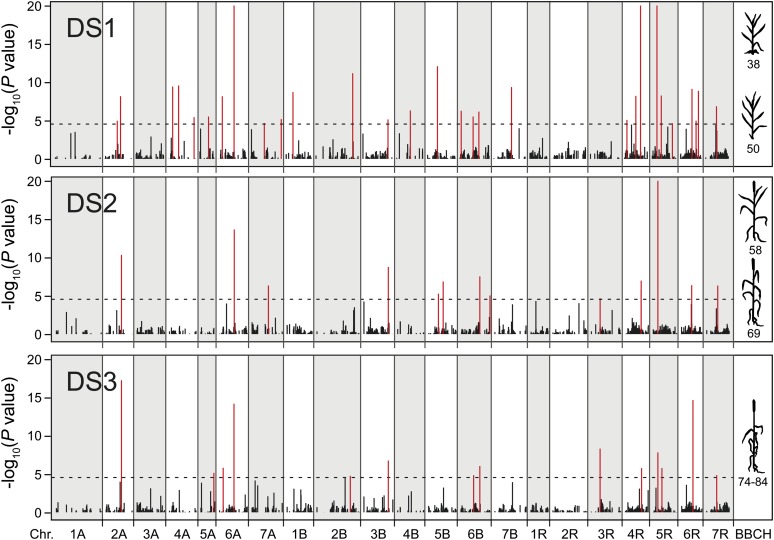
Quantitative trait loci for developmental stage at three time points (DS1−DS3). The dashed horizontal line indicates the significance threshold and significantly associated markers are shown in red.

**Figure 3:**
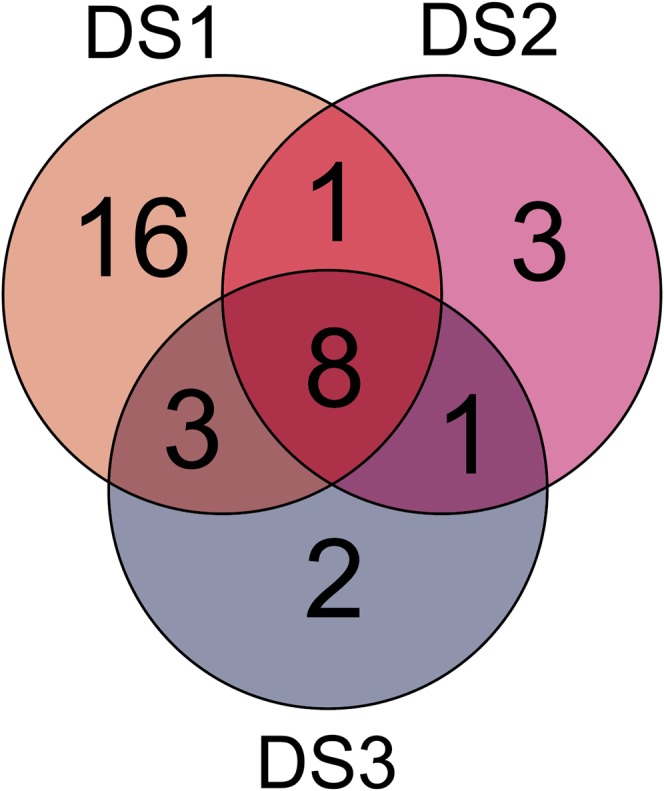
Venn diagram for developmental stage quantitative trait loci detected at three time points (DS1−DS3).

**Figure 4:**
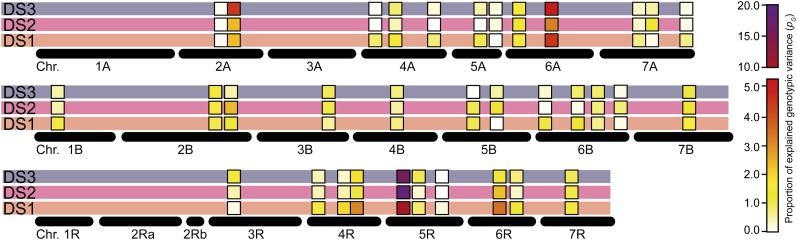
Temporal development of the contribution of the quantitative trait loci detected for any of the three time points to the proportion of explained genotypic variance for developmental stage (DS).

Employing association mapping in multiple families, we identified 29, 14, and 14 markers significantly associated with developmental stage at DS1, DS2, and DS3, respectively (Figure 2, Table 1, and Table S2). Interestingly, we identified eight markers that were found to be associated with developmental stage at all three time points, whereas the other markers were only detected at one or two of the time points (Figure 3). These markers explained 73.9% of the genotypic variance for DS1, 52.1% for DS2, and 57.4% for DS3. The proportion of genotypic variance (*p_G_*) explained by single markers ranged from 0.1 to 13.0% for DS1, from 0.1 to 18.3% for DS2, and from 0.2 to 17.4% for DS3 (Table S2). Major QTL explaining more than 3% of the genotypic variance were identified on chromosomes 6A, 5R, and 6R for DS1; on 4R and 5R for DS2; and on 2A, 6A, and 5R for DS3. The six parental lines showed differences in their allele composition at the detected QTL, despite their rather similar developmental stage at each of the three time points (Table S2). Consistent with the finding of time point-specific as well as overlapping QTL, we observed temporal dynamics of the contribution of these QTL regions to adult plant phenotypic development (Figure 4). Some QTL regions contributed to a similar extent throughout development, whereas for others the contribution to the genotypic variance was temporally defined. In line with these findings, we also detected QTL significantly associated with the developmental progression from one time point to another (Table S3 and Figure S2).

**Table 1:** Results of QTL mapping and fivefold cross-validation for developmental stage (DS) at three time points

	DS1	DS2	DS3
QTL_DS_	29	14	14
*p*_G-DS_	73.9	52.1	57.4
QTL_ES_	21.8	12.2	10.5
*p*_G-ES_	68.4	57.7	64.3
*p*_G-TS_	42.9	35.2	40.6
Relative bias	37.3	39.0	36.9

Number of detected quantitative trait loci (QTL_DS_), proportion of genotypic variance (%) explained by the detected QTL across all families in the data set (*p*_G-DS_), number of QTL (QTL_ES_), and proportion of genotypic variance averaged over estimation sets (*p*_G-ES_) and averaged over test sets (*p*_G-TS_), and relative bias (%) in the estimation of *p*_G_.

We employed a fivefold cross-validation approach to obtain asymptotically unbiased estimates of the proportion of genotypic variance explained by the detected markers. The cross-validated *p_G_* still ranged between 35.2% for DS2 to 42.9% for DS1 (Table 1). The obtained QTL frequency distributions revealed that most major QTL were identified in the majority of the runs whereas some other markers were only identified in few of the runs (Figure S3).

**Figure 5:**
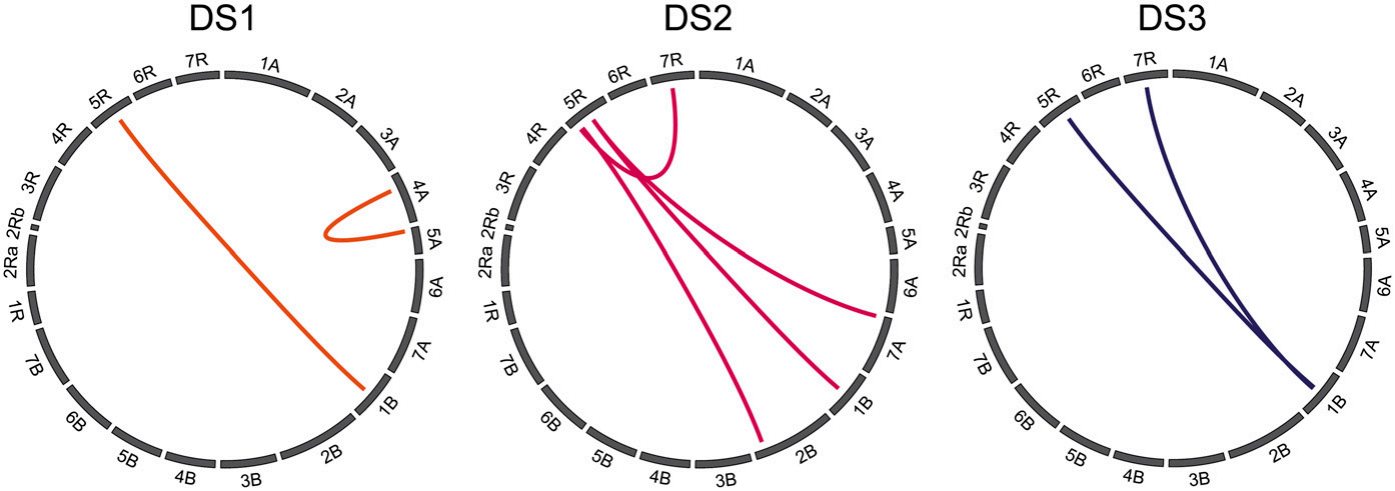
Epistatic quantitative trait loci for developmental stage at three time points (DS1−DS3).

We next performed a full two-dimensional scan for epistatic QTL at each of the three developmental stages, which revealed two epistatic interactions for DS1 and DS3 and four for DS2 (Figure 5). Of these, one epistatic QTL involving chromosomes 1B and 5R was identified at all three time points. The *p_G_* of these epistatic QTL ranged between 0.0 and 3.5%.

## Discussion

The progression of adult plant development affects traits such as flowering time or ripening and thus has consequences for many agronomically important traits, including adaptation and grain yield. The developmental stage of a plant is a plastic trait that undergoes dynamic temporal changes. Nevertheless, attempts to decipher the underlying genetics have thus far concentrated on single time points, mainly flowering time. In this study, we used a large mapping population and three time points to investigate the temporal dynamics of the genetic architecture during adult plant development.

### Phenotypic progression of adult plant development

Developmental stage was assessed in the mapping population at three time points when the majority of the plants were at the BBCH stages 49, 69, and 81, corresponding to the stages of awns becoming visible, late flowering, and very early dough development, respectively (Lancashire *et al.* 1991). The heritability was high for all three time points but highest for DS1, the first evaluated time point for which also the highest ratio of genotypic to genotype-by-environment interaction variance was observed (Table 1). This may indicate a stronger contribution of genotype-by-environment interactions at the later developmental stages.

The parents of the families were very similar in their developmental stage at each of the three time points (Figure 1). Nevertheless, we observed trangressive segregation, suggesting that the parents have all been adapted to the Central European environment by breeding but achieve this by different underlying genetics. The DH lines transgressing their respective parents suggest that the parents carry in part complementary QTL alleles that are newly combined in the progeny (Figure 1). The observed trait distributions indicate that adult plant development is a complex trait controlled by multiple loci and that different alleles for these loci are present in elite triticale germplasm. This allelic diversity is the foundation for the phenotypic plasticity that can be used in breeding programs. The data set under study thus represents an excellent resource to unravel the genetic basis of adult plant development in triticale as a representative for small grain cereals.

### Detection of QTL regions for developmental stage

The progression of developmental stage in small grain cereals is likely affected by a number of pathways and thus potentially also by the major QTL underlying plant height (*Rht* genes), vernalization (*Vrn* genes), or photoperiod (*Ppd*). The semi-dwarf *Reduced height* (*Rht*)*-1* homeologous loci from wheat are located on the group 4 chromosomes of the different genomes (Wilhelm *et al.* 2013). In the mapping population underlying this study, the major plant height gene *Ddw1* (Korzun *et al.* 1996; Börner *et al.* 1999), located on the rye chromosome 5R has been shown to segregate and to exhibit a strong effect on plant height (Alheit *et al.* 2014). *Photoperiod* (*Ppd*) homeologs are located on group 2 chromosomes and in wheat *Ppd-D1* has been shown to possess the strongest effect on photoperiod insensitivity and thus the transition from vegetative to generative growth (Beales *et al.* 2007). The homeologs of the three *Vernalization* (*Vrn*) genes are located on chromosomes 5 (*Vrn1*), 4 or 5 (*Vrn2*), and 7 (*Vrn3*), respectively (Yan *et al.* 2003, 2004, 2006). We identified QTL for all three time points which cross-validated still explained a considerable proportion of genotypic variance of approximately 40% (Table 1). The identified major QTL for the different time points, explaining more than 3% of the genotypic variance, were located on chromosomes 2A, 6A, 4R, 5R, and 6R (Table S2). The major QTL on chromosome 2A identified to affect plant development might correspond to a *Ppd* homeolog and the QTL on 5B to *Vrn-1*, and both may be identified due to an accelerated transition to the generative phase by certain alleles at these loci. The other identified major QTL do not appear to correspond to any of the abovementioned major genes and therefore may represent other pathways affecting plant development. We have previously performed a QTL mapping in this population for plant height (Alheit *et al.* 2014) and 9 of the 12 plant height QTL regions were identified in this study to affect developmental stage. Although these QTL were not major QTL, this finding illustrates the interrelations between plant height and adult plant development. As discussed previously, the evaluation of the allele composition at the detected QTL for the six parental lines indeed revealed differences among them consistent with the observed strong variation within the families.

Epistasis refers to interactions between two or more loci in the genome and has recently been shown to contribute to the genetic control of complex traits in both triticale parents, wheat and rye (Li *et al.* 2011; Reif *et al.* 2011b). In addition to main effect QTL, we also identified few epistatic QTL for all three time points in development (Figure 5). In general, however, identified epistatic interactions do not allow to infer the biological mechanisms of the gene interactions (Carlborg and Haley 2004). The limited number of detected epistatic QTL may be misleading because the power to detect them depends more on population size than the power for the detection of main effect QTL. Thus, a higher number of epistatic QTL may be involved but remained undetected despite the comparably large size of the mapping population. Our results thus illustrate that epistatic interactions are a component of the genetic architecture underlying adult plant development in triticale.

### Temporal dynamics underlying the genetic control

Chromosomal regions harboring QTL were identified for all three time points but more for DS1 than for the later two time points (Figure 2 and 3). DS1 corresponded to the developmental stage when the awns became visible. This may indicate that development until that stage is shaped predominantly by QTL with effects large enough to be detected by QTL mapping, whereas the subsequent development might require more fine-tuning orchestrated by smaller effect QTL that escape detection. Interestingly, some QTL were detected at all three time points, whereas others were only identified at one or two time points. As illustrated by the Manhattan plots (Figure 3), this temporal contribution of QTL to adult plant development was not simply an artifact of these QTL being just above or below the significance threshold at the different time points; rather, it shows that some QTL are active and contribute to developmental progression during a longer period of time encompassing several developmental stages whereas other QTL act in a more timely restricted manner. This conclusion was further substantiated by the varying contribution of the identified QTL regions to the genotypic variance (Figure 4). An alternative approach to assess the genetics underlying dynamic traits is termed functional mapping, where genetic differences in growth trajectories during trait development are used for QTL mapping (Ma *et al.* 2002; Zhao *et al.* 2005; Wu and Lin 2006). This, however, requires a much higher number of time points as used in this study, but as illustrated by our results warrants future research for adult plant development in cereals.

Furthermore, we observed that for some chromosomal regions identified as QTL at more than one time point, the sign of the additive effect changed between time points (*e.g.*, QTL on chromosome 6A) (Table S2). This was mirrored by QTL detected for the same regions for the developmental progression between the stages when the change in sign of the QTL effects occurred (Table S2, Table S3, and Figure S2). This finding may indicate that certain QTL alleles accelerate or decelerate developmental progression in a developmental stage-specific manner. Such information could be useful in applied plant breeding to tailor varieties which, for example, have an extended grain filling phase to maximize yield potential. Taken together, the plasticity of adult plant development in triticale is mirrored by dynamic genetic patterns of regulation.

In this study we used a large triticale mapping population, phenotyped at different time points, to investigate the genetic control underlying adult plant development. Our results show that both main and epistatic QTL contribute to the genetic architecture of developmental stage at all three time points. By considering more than just a single time point in development we demonstrate that development during the reproductive phase in triticale is controlled by dynamic patterns of genetic regulation. This confirms that a temporal assessment, as facilitated for example by precision phenotyping platforms (Busemeyer *et al.* 2013b), is of paramount importance to study the changes in the genetic control underlying dynamic traits. In a wider context, our discoveries on the dynamic genetic control underlying triticale development are likely to have broad relevance to other crops as well as other plant species in general, as adult plant development likely shares a similar dynamic genetic pattern of regulation.

## Supplementary Material

Supporting Information
