# Supplementary material for: Adult Plant Development in Triticale (× Triticosecale Wittmack) Is Controlled by Dynamic Genetic Patterns of Regulation
Source: G3 (Bethesda). 2014 Sep 1;4(9):1585–91. doi: 10.1534/g3.114.012989 (PMC4169150; doi:10.1534/g3.114.012989)
Supplement: Supporting Information [file supp_4_9_1585__index.html]

Supporting Information 

# Adult Plant Development in Triticale (× *Triticosecale* Wittmack) Is Controlled by Dynamic Genetic Patterns of Regulation

## Supporting Information for W?rschum *et al.*, 2014

**Files in this Data Supplement:**

- Supporting Information - Figures S1-S3, File S1, and Tables S1-S3 (PDF, 309 KB)
- Figure S1 - Principal coordinate analysis of the four families and the six parents based on modified Rogers' distance estimates (PDF, 309 KB)
- Figure S2 - QTL detected for the progression in developmental stage between the three time points (DS1, DS2, DS3) (PDF, 105 KB)
- Figure S3 - Frequency distributions from the fivefold cross-validation for the QTL detected for developmental stage at three time points (DS1-DS3) (PDF, 100 KB)
- Table S1 - Summary statistics for developmental stage at three time points (DS1-DS3) (PDF, 98 KB)
- Table S2 - QTL detected for developmental stage at three time points (DS1-DS3) (PDF, 101 KB)
- Table S3 - QTL detected for the progression in developmental stage between the three time points (DS1, DS2, DS3) (PDF, 84 KB)
- File S1 - Phenotypic and genotypic data underlying the study and P values from the genome scans (.xlsx, 4.1 MB)
