# Supplementary material for: Adult Plant Development in Triticale (× Triticosecale Wittmack) Is Controlled by Dynamic Genetic Patterns of Regulation
Source: G3 (Bethesda). 2014 Sep 1;4(9):1585–91. doi: 10.1534/g3.114.012989 (PMC4169150; doi:10.1534/g3.114.012989)
Supplement: Supporting Information [file supp_4.9.1585_012989SI.pdf]

G3·Genes | Genomes | Genetics

Adult plant development in triticale (× *Triticosecale* Wittmack) is controlled by dynamic genetic patterns of regulation

Tobias Würschum*[,1], Wenxin Liu[§], Katharina V. Alheit*, Matthew R. Tucker[†], Manje Gowda*, Elmar A. Weissmann[‡], Volker Hahn*, Hans Peter Maurer*

* State Plant Breeding Institute, University of Hohenheim, 70599 Stuttgart, Germany

[§] Crop Genetics and Breeding Department, China Agricultural University, 100193 Beijing, China

[†] ARC Centre of Excellence for Plant Cell Walls, University of Adelaide, Waite Campus, Urrbrae SA 5064, Australia

[‡] Saatzucht Dr. Hege GbR Domäne Hohebuch, 74638 Waldenburg, Germany

[1] Corresponding author: State Plant Breeding Institute, University of Hohenheim, 70599 Stuttgart, Germany. Email: tobias.wuerschum@uni-hohenheim.de

Corresponding author:
Tobias Würschum
Adress: State Plant Breeding Institute, University of Hohenheim, 70599 Stuttgart, Germany.
Phone: +49-711-45922687
E-mail: tobias.wuerschum@uni-hohenheim.de

DOI: 10.1534/g3.114.012989

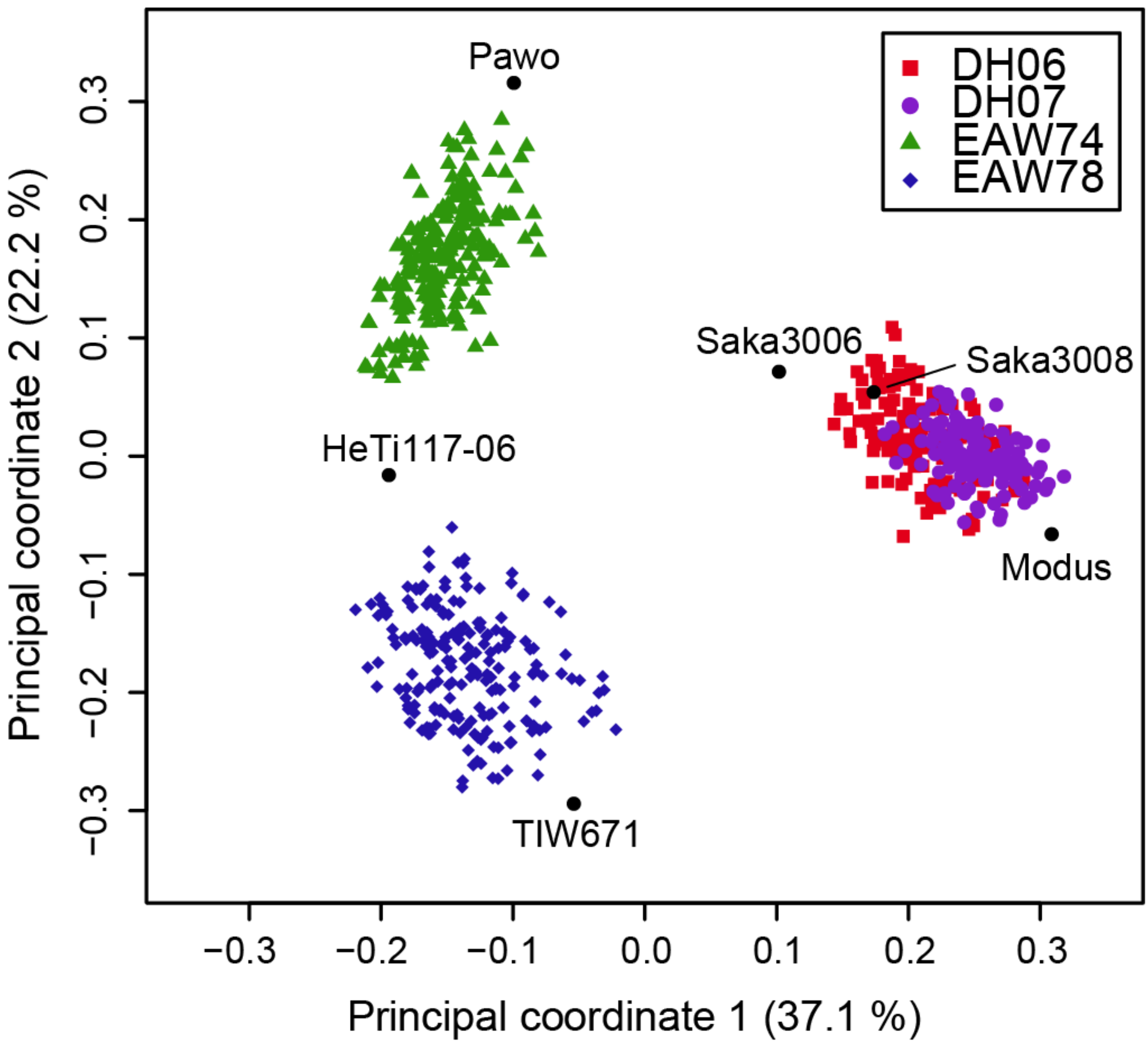

Figure S1 Principal coordinate analysis of the four families and the six parents based on modified Rogers' distance estimates. Percentages in parentheses refer to the proportion of variance explained by the principal coordinate.

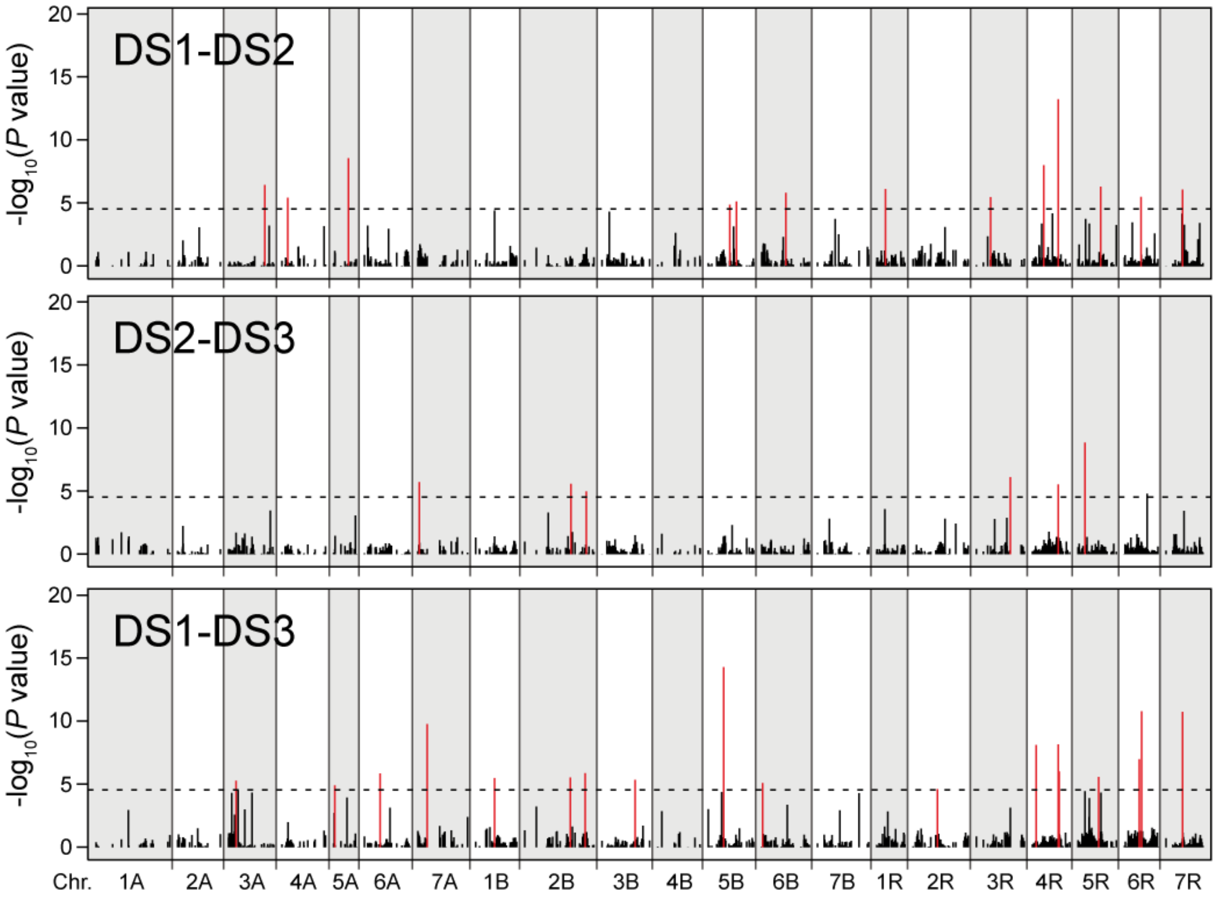

Figure S2 QTL detected for the progression in developmental stage between the three time points (DS1, DS2, DS3).

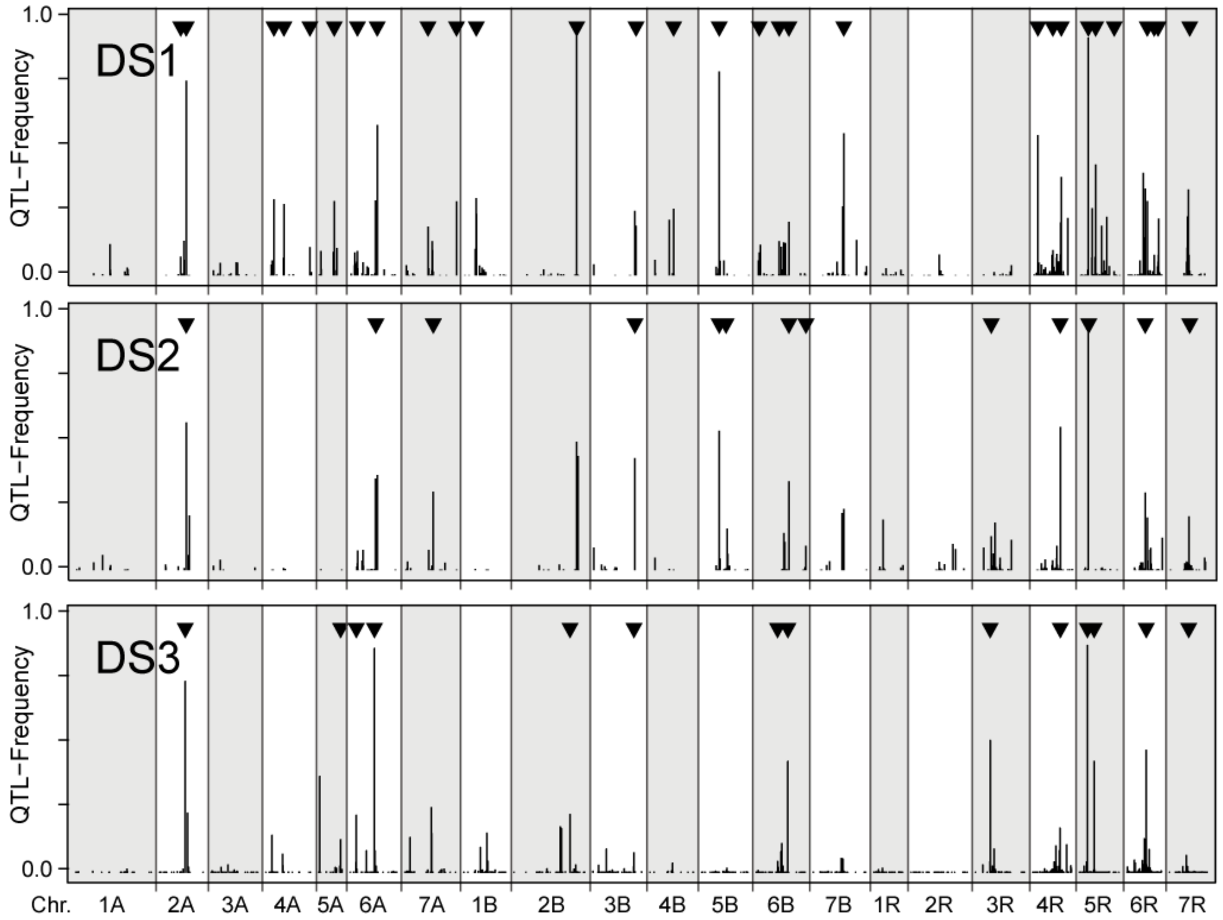

Figure S3 Frequency distributions from the fivefold cross-validation for the QTL detected for developmental stage at three time points (DS1-DS3). The arrowheads indicate the positions of QTL detected with the full data set.

File S1

Phenotypic and genotypic data underlying the study and P values from the genome scans.

Available for download as an Excel file at http://www.g3journal.org/lookup/suppl/doi:10.1534/g3.114.012989/-/DC1

Table S1 Summary statistics for developmental stage at three time points (DS1-DS3).

	DS1	DS2	DS3
Min	38.0	58.6	74.6
Mean	44.0	67.0	80.9
Max	49.6	69.4	84.3
σ_G^2	4.48**	3.25**	1.96**
σ_{GxE}^2	1.01**	1.45**	0.62**
σ_e^2	1.53	1.28	3.56
h^2	0.90	0.85	0.72

Genotypic variance (σ_G^2), genotype-by-environment interaction variance (σ_{GxE}^2), error variance (σ_e^2) and heritability (h^2). **significant at $P < 0.01$

Table S2 QTL detected for developmental stage at three time points (DS1-DS3). Chromosome, position (in cM), QTL-effect and proportion of genotypic variance explained by the QTL (in percent). Markers within an arbitrarily defined 10 cM interval were considered as identical QTL. The allele status of the six parents at these markers is also provided.

Marker	Chr.	Pos.	DS1		DS2		DS3		Modus	Saka 3006	Saka 3008	HeTi 117-06	Pawo	TIW671
			QTL-effect	p_G	QTL-effect	p_G	QTL-effect	p_G						
wPt-7026	2A	48.9	-0.23	0.2					0	0	1	0	0	0
wPt-3114	2A	62.1	-0.30	2.2	-0.30	2.9	-0.32	4.9	1	0	0	1	0	0
tPt-512917	4A	17.1	0.43	1.7					0	1	1	1	1	1
wPt-5857	4A	41.1	-0.31	0.6					0	1	1	1	1	0
wPt-7391	4A	103.8	-0.19	0.7					1	0	0	1	0	0
wPt-2329	5A	34.8	0.29	0.9					1	0	0	1	1	1
wPt-7255	5A	52.5					-0.22	0.7	0	0	0	0	0	1
wPt-4017	6A	14.5					0.15	1.1	1	0	0	0	0	1
wPt-7330	6A	14.7	0.27	1.0					1	0	0	0	0	1
wPt-0902	6A	58.2					-0.50	5.0	1	1	1	1	1	0
wPt-2077	6A	59.2			0.69	2.7			1	1	1	0	0	1
tPt-4209	6A	62.4	0.46	4.1					1	1	1	0	1	1
wPt-7785	7A	53.2	-0.18	0.6					0	1	1	1	0	0
wPt-7299	7A	66.0			0.35	1.5			0	1	0	0	0	1
wPt-0494	7A	121.9	0.21	0.9					1	0	0	0	1	1
wPt-5003	1B	27.7	0.45	1.8					0	1	1	0	0	0
wPt-9958	2B	134.9					0.20	1.9	1	0	0	1	0	0
tPt-1663	2B	148.6	0.39	2.9					1	1	1	1	0	0
wPt-9422	3B	98.7			-0.27	0.4	-0.15	0.2	0	1	0	1	0	0
tPt-513153	3B	101.4	0.15	0.4					1	0	0	0	1	1
wPt-6016	4B	54.9	0.33	0.7					1	1	1	1	0	1
wPt-1548	5B	39.9	0.52	1.5	0.43	1.6			1	1	1	1	1	0
wPt-1733	5B	59.1			0.45	1.3			0	0	0	0	1	0
wPt-3304	6B	5.0	0.32	0.6					1	0	1	1	0	1
wPt-7426	6B	51.5					-0.20	1.1	0	0	0	1	0	0
wPt-2400	6B	53.2	0.20	0.7					0	1	1	0	1	1
wPt-3581	6B	76.5	0.33	1.5	0.33	1.3	0.30	1.4	0	1	1	1	1	1
rPt-505542	6B	117.7			0.22	1.1			0	1	1	1	0	0
wPt-1149	7B	71.2	0.52	2.8					1	1	1	1	0	1
rPt-507396	3R	35.2					-0.33	0.9	1	1	1	1	1	0
rPt-402572	3R	35.4			-0.22	0.8			1	0	0	1	1	0
rPt-389770	4R	9.9	0.60	1.5					1	0	1	-	-	-
rPt-509552	4R	45.9	0.48	2.0					0	0	0	0	1	0
rPt-509321	4R	63.4			-0.49	3.1			1	0	0	1	0	0
rPt-389618	4R	63.8			-0.50	0.1			0	1	1	0	1	1
rPt-506436	4R	65.0	0.51	2.3					1	0	0	0	0	1
rPt-410866	4R	65.4					-0.22	1.9	0	1	0	1	0	0
rPt-399681	5R	18.9	1.33	13.0	1.45	18.3	1.04	17.4	0	0	0	1	1	0
rPt-402367	5R	35.2					0.30	1.8	0	0	0	1	0	1
rPt-508041	5R	36.5	0.38	0.7					0	0	0	1	0	1
rPt-505265	5R	81.3	0.09	0.1					1	0	0	1	1	1
rPt-507562	6R	41.2			-0.36	2.6			0	0	1	1	0	0
rPt-401125	6R	46.2	-0.54	3.5			-0.24	1.4	0	0	0	1	0	0
rPt-508379	6R	62.8	0.24	0.5					1	0	0	0	1	1
rPt-390698	6R	72.3	0.43	0.9					0	1	1	1	1	0
rPt-400878	7R	40.4					0.21	1.1	0	1	0	1	1	0
rPt-390741	7R	43.3	-0.35	1.4					1	0	1	0	0	1
rPt-401147	7R	44.6			0.39	0.8			1	1	0	1	1	1

Table S3 QTL detected for the progression in developmental stage between the three time points (DS1, DS2, DS3). Chromosome, position (in cM), QTL-effect and proportion of genotypic variance explained by the QTL (in percent). Markers within an arbitrarily defined 10 cM interval were considered as identical QTL.

Marker	Chr.	Position	DS1-DS2		DS2-DS3		DS1-DS3	
			QTL-effect	p_G	QTL-effect	p_G	QTL-effect	p_G
wPt-2866	3A	18.8					0.03	0.0
wPt-9761	3A	87.7	-0.34	2.2				
wPt-8657	4A	12.6	0.16	1.9				
wPt-3334	5A	2.5					0.32	1.3
wPt-2329	5A	34.8	-0.23	0.0				
wPt-0259	6A	38.1					0.24	0.2
wPt-5101	7A	3.3			-0.45	6.4		
wPt-3523	7A	22.8					-0.36	3.5
wPt-7066	1B	46.4					0.32	4.2
wPt-8460	2B	112.0					-0.20	2.4
rPt-506241	2B	113.9			-0.26	1.3		
tPt-1663	2B	148.6					-0.20	1.0
wPt-9274	2B	151.7			-0.21	1.6		
wPt-345354	3B	81.8					0.21	1.6
wPt-8106	5B	38.0					-0.27	1.3
wPt-5604	5B	51.0	-0.11	0.4				
tPt-1253	5B	67.9	-0.27	2.8				
wPt-8641	6B	2.6					-0.21	0.2
wPt-8721	6B	57.8	0.27	2.1				
rPt-506392	1R	19.9	0.07	0.0				
tPt-513832	2Ra	60.4					-0.38	1.3
rPt-507396	3R	35.2	-0.10	0.0				
rPt-507569	3R	84.0			0.27	3.7		
rPt-389770	4R	9.9					-0.42	0.3
rPt-401450	4R	28.2	-0.57	7.4				
rPt-505225	4R	63.8	0.31	0.9				
rPt-505489	4R	63.9			-0.07	0.3		
rPt-411490	4R	63.9					-0.27	3.9
rPt-390324	4R	66.3					0.36	4.0
rPt-509721	5R	17.0			-0.28	10.6		
rPt-399462	5R	51.1					0.38	3.6
rPt-400368	5R	55.6	0.11	0.3				
tPt-513060	6R	36.2					-0.42	6.2
rPt-8205	6R	40.6	-0.10	0.7				
rPt-507115	6R	41.7					0.25	2.9
rPt-402205	6R	55.7			0.47	4.2		
rPt-390750	7R	40.6					-0.34	0.2
tPt-514019	7R	41.0	0.07	0.5				
