# Supplementary material for: Adult Plant Development in Triticale (× Triticosecale Wittmack) Is Controlled by Dynamic Genetic Patterns of Regulation
Source: G3 (Bethesda). 2014 Sep 1;4(9):1585–91. doi: 10.1534/g3.114.012989 (PMC4169150; doi:10.1534/g3.114.012989)
Supplement: Supporting Information [file supp_4.9.1585_FigureS1.pdf]

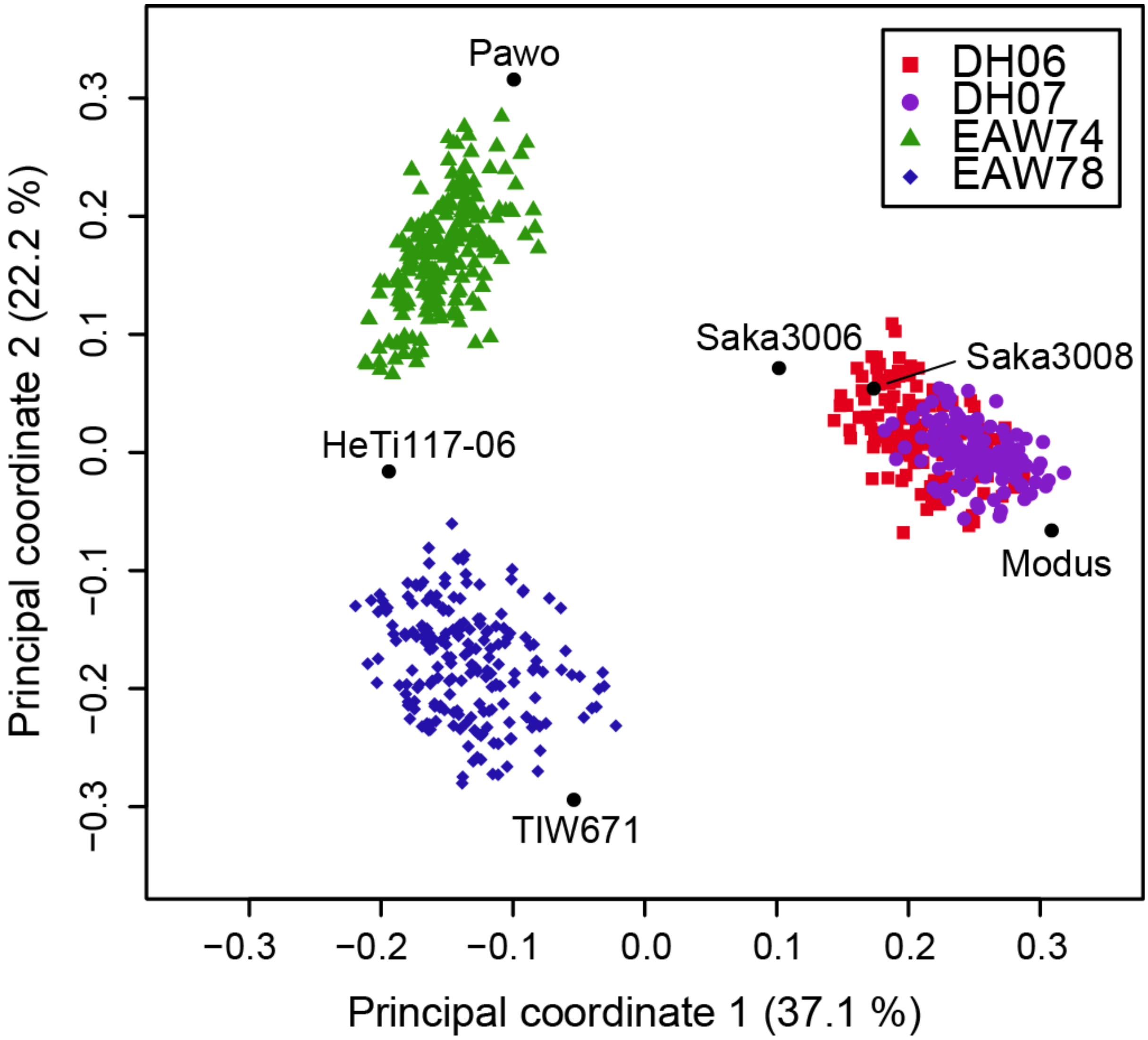

Figure S1 Principal coordinate analysis of the four families and the six parents based on modified Rogers' distance estimates. Percentages in parentheses refer to the proportion of variance explained by the principal coordinate.
