# Supplementary material for: Adult Plant Development in Triticale (× Triticosecale Wittmack) Is Controlled by Dynamic Genetic Patterns of Regulation
Source: G3 (Bethesda). 2014 Sep 1;4(9):1585–91. doi: 10.1534/g3.114.012989 (PMC4169150; doi:10.1534/g3.114.012989)
Supplement: Supporting Information [file supp_4.9.1585_FigureS3.pdf]

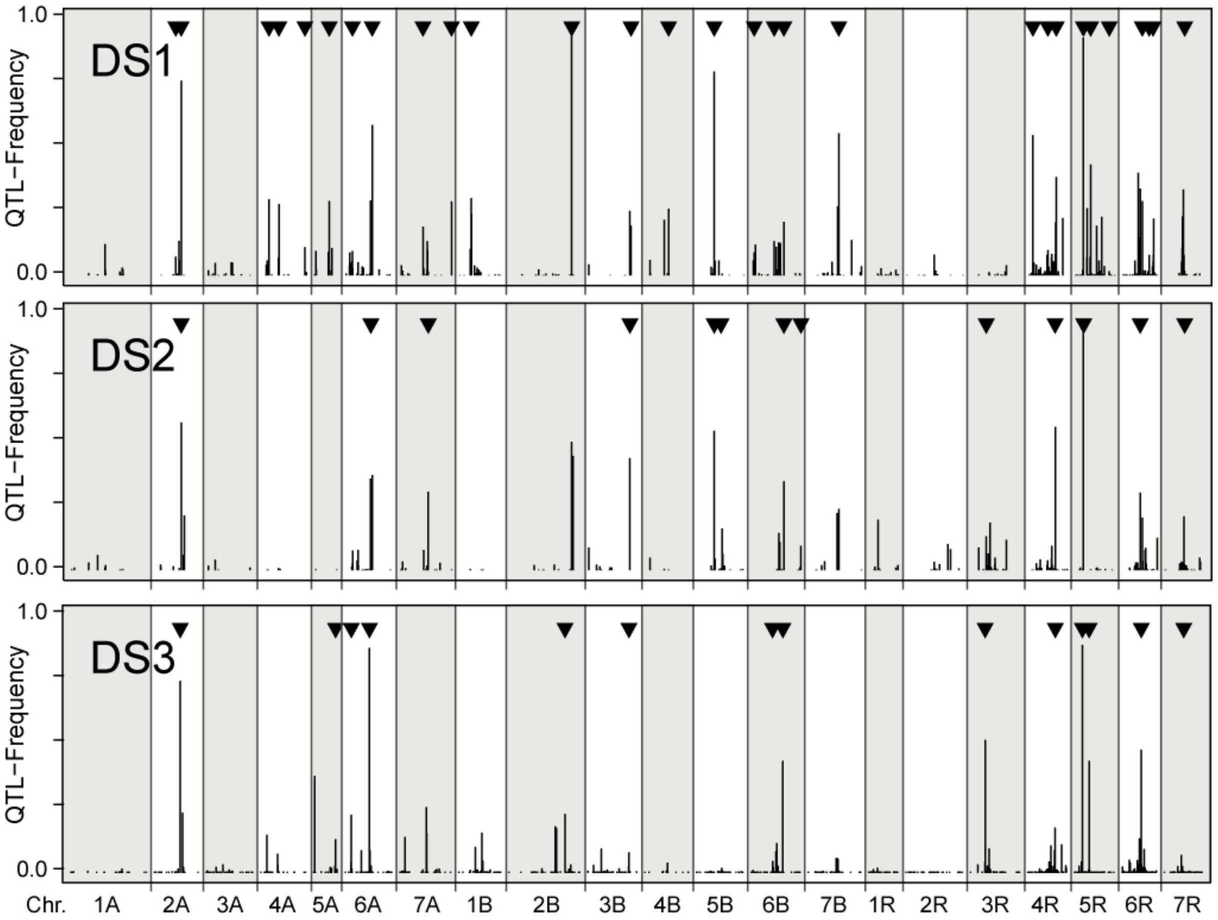


**Figure S3** Frequency distributions from the fivefold cross-validation for the QTL detected for developmental stage at three time points (DS1-DS3). The arrowheads indicate the positions of QTL detected with the full data set.
