# Supplementary material for: Adult Plant Development in Triticale (× Triticosecale Wittmack) Is Controlled by Dynamic Genetic Patterns of Regulation
Source: G3 (Bethesda). 2014 Sep 1;4(9):1585–91. doi: 10.1534/g3.114.012989 (PMC4169150; doi:10.1534/g3.114.012989)
Supplement: Supporting Information [file supp_4.9.1585_TableS1.pdf]

Table S1 Summary statistics for developmental stage at three time points (DS1-DS3).

	DS1	DS2	DS3
Min	38.0	58.6	74.6
Mean	44.0	67.0	80.9
Max	49.6	69.4	84.3
σ_G^2	4.48**	3.25**	1.96**
σ_{GxE}^2	1.01**	1.45**	0.62**
σ_e^2	1.53	1.28	3.56
h^2	0.90	0.85	0.72

Genotypic variance (σ_G^2), genotype-by-environment interaction variance (σ_{GxE}^2), error variance (σ_e^2) and heritability (h^2). **significant at $P < 0.01$
