# Supplementary material for: Adult Plant Development in Triticale (× Triticosecale Wittmack) Is Controlled by Dynamic Genetic Patterns of Regulation
Source: G3 (Bethesda). 2014 Sep 1;4(9):1585–91. doi: 10.1534/g3.114.012989 (PMC4169150; doi:10.1534/g3.114.012989)
Supplement: Supporting Information [file supp_4.9.1585_TableS2.pdf]

Table S2 QTL detected for developmental stage at three time points (DS1-DS3). Chromosome, position (in cM), QTL-effect and proportion of genotypic variance explained by the QTL (in percent). Markers within an arbitrarily defined 10 cM interval were considered as identical QTL. The allele status of the six parents at these markers is also provided.

Marker	Chr.	Pos.	DS1		DS2		DS3		Modus	Saka 3006	Saka 3008	HeTi 117-06	Pawo	TIW671
			QTL-effect	p_G	QTL-effect	p_G	QTL-effect	p_G						
wPt-7026	2A	48.9	-0.23	0.2					0	0	1	0	0	0
wPt-3114	2A	62.1	-0.30	2.2	-0.30	2.9	-0.32	4.9	1	0	0	1	0	0
tPt-512917	4A	17.1	0.43	1.7					0	1	1	1	1	1
wPt-5857	4A	41.1	-0.31	0.6					0	1	1	1	1	0
wPt-7391	4A	103.8	-0.19	0.7					1	0	0	1	0	0
wPt-2329	5A	34.8	0.29	0.9					1	0	0	1	1	1
wPt-7255	5A	52.5					-0.22	0.7	0	0	0	0	0	1
wPt-4017	6A	14.5					0.15	1.1	1	0	0	0	0	1
wPt-7330	6A	14.7	0.27	1.0					1	0	0	0	0	1
wPt-0902	6A	58.2					-0.50	5.0	1	1	1	1	1	0
wPt-2077	6A	59.2			0.69	2.7			1	1	1	0	0	1
tPt-4209	6A	62.4	0.46	4.1					1	1	1	0	1	1
wPt-7785	7A	53.2	-0.18	0.6					0	1	1	1	0	0
wPt-7299	7A	66.0			0.35	1.5			0	1	0	0	0	1
wPt-0494	7A	121.9	0.21	0.9					1	0	0	0	1	1
wPt-5003	1B	27.7	0.45	1.8					0	1	1	0	0	0
wPt-9958	2B	134.9					0.20	1.9	1	0	0	1	0	0
tPt-1663	2B	148.6	0.39	2.9					1	1	1	1	0	0
wPt-9422	3B	98.7			-0.27	0.4	-0.15	0.2	0	1	0	1	0	0
tPt-513153	3B	101.4	0.15	0.4					1	0	0	0	1	1
wPt-6016	4B	54.9	0.33	0.7					1	1	1	1	0	1
wPt-1548	5B	39.9	0.52	1.5	0.43	1.6			1	1	1	1	1	0
wPt-1733	5B	59.1			0.45	1.3			0	0	0	0	1	0
wPt-3304	6B	5.0	0.32	0.6					1	0	1	1	0	1
wPt-7426	6B	51.5					-0.20	1.1	0	0	0	1	0	0
wPt-2400	6B	53.2	0.20	0.7					0	1	1	0	1	1
wPt-3581	6B	76.5	0.33	1.5	0.33	1.3	0.30	1.4	0	1	1	1	1	1
rPt-505542	6B	117.7			0.22	1.1			0	1	1	1	0	0
wPt-1149	7B	71.2	0.52	2.8					1	1	1	1	0	1
rPt-507396	3R	35.2					-0.33	0.9	1	1	1	1	1	0
rPt-402572	3R	35.4			-0.22	0.8			1	0	0	1	1	0
rPt-389770	4R	9.9	0.60	1.5					1	0	1	-	-	-
rPt-509552	4R	45.9	0.48	2.0					0	0	0	0	1	0
rPt-509321	4R	63.4			-0.49	3.1			1	0	0	1	0	0
rPt-389618	4R	63.8			-0.50	0.1			0	1	1	0	1	1
rPt-506436	4R	65.0	0.51	2.3					1	0	0	0	0	1
rPt-410866	4R	65.4					-0.22	1.9	0	1	0	1	0	0
rPt-399681	5R	18.9	1.33	13.0	1.45	18.3	1.04	17.4	0	0	0	1	1	0
rPt-402367	5R	35.2					0.30	1.8	0	0	0	1	0	1
rPt-508041	5R	36.5	0.38	0.7					0	0	0	1	0	1
rPt-505265	5R	81.3	0.09	0.1					1	0	0	1	1	1
rPt-507562	6R	41.2			-0.36	2.6			0	0	1	1	0	0
rPt-401125	6R	46.2	-0.54	3.5			-0.24	1.4	0	0	0	1	0	0
rPt-508379	6R	62.8	0.24	0.5					1	0	0	0	1	1
rPt-390698	6R	72.3	0.43	0.9					0	1	1	1	1	0
rPt-400878	7R	40.4					0.21	1.1	0	1	0	1	1	0
rPt-390741	7R	43.3	-0.35	1.4					1	0	1	0	0	1
rPt-401147	7R	44.6			0.39	0.8			1	1	0	1	1	1
