# Supplementary material for: Adult Plant Development in Triticale (× Triticosecale Wittmack) Is Controlled by Dynamic Genetic Patterns of Regulation
Source: G3 (Bethesda). 2014 Sep 1;4(9):1585–91. doi: 10.1534/g3.114.012989 (PMC4169150; doi:10.1534/g3.114.012989)
Supplement: Supporting Information [file supp_4.9.1585_TableS3.pdf]

Table S3 QTL detected for the progression in developmental stage between the three time points (DS1, DS2, DS3). Chromosome, position (in cM), QTL-effect and proportion of genotypic variance explained by the QTL (in percent). Markers within an arbitrarily defined 10 cM interval were considered as identical QTL.

Marker	Chr.	Position	DS1-DS2		DS2-DS3		DS1-DS3	
			QTL-effect	p_G	QTL-effect	p_G	QTL-effect	p_G
wPt-2866	3A	18.8					0.03	0.0
wPt-9761	3A	87.7	-0.34	2.2				
wPt-8657	4A	12.6	0.16	1.9				
wPt-3334	5A	2.5					0.32	1.3
wPt-2329	5A	34.8	-0.23	0.0				
wPt-0259	6A	38.1					0.24	0.2
wPt-5101	7A	3.3			-0.45	6.4		
wPt-3523	7A	22.8					-0.36	3.5
wPt-7066	1B	46.4					0.32	4.2
wPt-8460	2B	112.0					-0.20	2.4
rPt-506241	2B	113.9			-0.26	1.3		
tPt-1663	2B	148.6					-0.20	1.0
wPt-9274	2B	151.7			-0.21	1.6		
wPt-345354	3B	81.8					0.21	1.6
wPt-8106	5B	38.0					-0.27	1.3
wPt-5604	5B	51.0	-0.11	0.4				
tPt-1253	5B	67.9	-0.27	2.8				
wPt-8641	6B	2.6					-0.21	0.2
wPt-8721	6B	57.8	0.27	2.1				
rPt-506392	1R	19.9	0.07	0.0				
tPt-513832	2Ra	60.4					-0.38	1.3
rPt-507396	3R	35.2	-0.10	0.0				
rPt-507569	3R	84.0			0.27	3.7		
rPt-389770	4R	9.9					-0.42	0.3
rPt-401450	4R	28.2	-0.57	7.4				
rPt-505225	4R	63.8	0.31	0.9				
rPt-505489	4R	63.9			-0.07	0.3		
rPt-411490	4R	63.9					-0.27	3.9
rPt-390324	4R	66.3					0.36	4.0
rPt-509721	5R	17.0			-0.28	10.6		
rPt-399462	5R	51.1					0.38	3.6
rPt-400368	5R	55.6	0.11	0.3				
tPt-513060	6R	36.2					-0.42	6.2
rPt-8205	6R	40.6	-0.10	0.7				
rPt-507115	6R	41.7					0.25	2.9
rPt-402205	6R	55.7			0.47	4.2		
rPt-390750	7R	40.6					-0.34	0.2
tPt-514019	7R	41.0	0.07	0.5				
